# Target attainment of intravenous lefamulin for treatment of acute bacterial skin and skin structure infections

**DOI:** 10.1093/jac/dkad401

**Published:** 2024-01-04

**Authors:** Wisse van Os, Markus Zeitlinger

**Affiliations:** Department of Clinical Pharmacology, Medical University of Vienna, Waehringer Guertel 18-20, 1090 Vienna, Austria; Department of Clinical Pharmacology, Medical University of Vienna, Waehringer Guertel 18-20, 1090 Vienna, Austria

## Abstract

**Objectives:**

Lefamulin is a pleuromutilin antibiotic approved for the treatment of community-acquired bacterial pneumonia (CABP). Its spectrum of activity, good penetration into soft tissues and low rates of cross-resistance also make lefamulin a potentially valuable option for treatment of acute bacterial skin and skin structure infections (ABSSSIs). A Phase 2 trial of lefamulin for ABSSSI indicated similar efficacy of 100 and 150 mg q12h IV dosing regimens. In the present study, the potential of lefamulin for this indication was further evaluated from a translational pharmacokinetic/pharmacodynamic perspective.

**Methods:**

PTA was determined for various dosages using Monte Carlo simulations of a population pharmacokinetic model of lefamulin in ABSSSI patients and preclinical exposure targets associated with bacteriostasis and a 1-log reduction in bacterial count. Overall target attainment against MSSA and MRSA was calculated using lefamulin MIC distributions.

**Results:**

Overall attainment of the bacteriostasis target was 94% against MSSA and 84% against MRSA for the IV dosage approved for CABP (150 mg q12h). Using the same target, for the 100 mg q12h regimen, overall target attainment dropped to 68% against MSSA and 50% against MRSA. Using the 1-log reduction target, overall target attainment for both regimens was <40%.

**Conclusions:**

Lefamulin at the currently approved IV dosage covers most *Staphylococcus aureus* isolates when targeting drug exposure associated with bacteriostasis, suggesting potential of lefamulin for the treatment of ABSSSIs. Lefamulin may not be appropriate in ABSSSI when rapid bactericidal activity is warranted.

## Introduction

Lefamulin is the first pleuromutilin antibiotic for systemic use in humans. It is approved for the treatment of community-acquired bacterial pneumonia (CABP), but its spectrum of activity^1^ and extensive penetration into soft tissues^2^ also render it a potentially interesting option for treatment of acute bacterial skin and skin structure infections (ABSSSIs). A Phase 2 trial in patients with ABSSSIs found similar clinical and microbiological success rates for lefamulin at IV dosages of 100 or 150 mg q12h, and vancomycin dosed at 1 g q12h IV, supporting development for this indication.^3^ The objective of this analysis was to evaluate the potential of lefamulin for ABSSSIs from a translational pharmacokinetic/pharmacodynamic (PK/PD) perspective by combining population PK model simulations, preclinical PK/PD targets and MIC distributions of *Staphylococcus aureus*, the pathogen responsible for the majority of skin and soft-tissue infections.^4^

## Methods

Monte Carlo simulations using a previously reported population PK model for lefamulin^5^ were performed using NONMEM (version 7.4; ICON plc, Gaithersburg, MD, USA). The model was developed using data from a Phase 2 study of lefamulin in patients with ABSSSIs and was based on 1167 concentrations from 129 patients. The final model was a three-compartment model with first-order elimination, non-linear protein binding and no covariate relationships.^5^ Using the typical parameter values and interindividual variability estimated in the model, free-drug plasma PK profiles were simulated for patients receiving 100 or 150 mg q12h by 1 h IV infusions (*n* = 10 000 patients per dose group). Alternative doses up to 1000 mg q12h were also simulated, assuming the model is valid for the full dose range. Integration of the concentration–time curves between 0 and 24 h after the first administration was performed directly in NONMEM to obtain free drug area under the curve (*f*AUC_0–24h_) values for each simulated patient. The *f*AUC_0–24h_/MIC ratio, which is the PK/PD index that correlates best with lefamulin effect,^6,7^ was calculated for different MIC values (0.008–32 mg/L) for each patient. The PK/PD targets used in this analysis were *f*AUC_0–24h_/MIC ratios of ≥14 or ≥33, associated with bacteriostasis and a 1-log reduction in bacterial count, respectively, over 24 h against *S. aureus* in a neutropenic murine thigh infection model,^7^ which is an appropriate translational model for soft-tissue infections.^8^ PTA was calculated as the percentage of simulated patients who achieved these PK/PD targets. To link the PTA results to coverage of *S. aureus*, lefamulin MIC distributions for MSSA and MRSA were obtained from EUCAST (*n* = 8015 and 5429 observations for MSSA and MRSA, respectively, as of September 2023).^9^ By weighting PTA for each MIC value over the relative frequency of that MIC occurring, the overall target attainment (OTA) was calculated.

## Results and discussion

**Figure 1.**
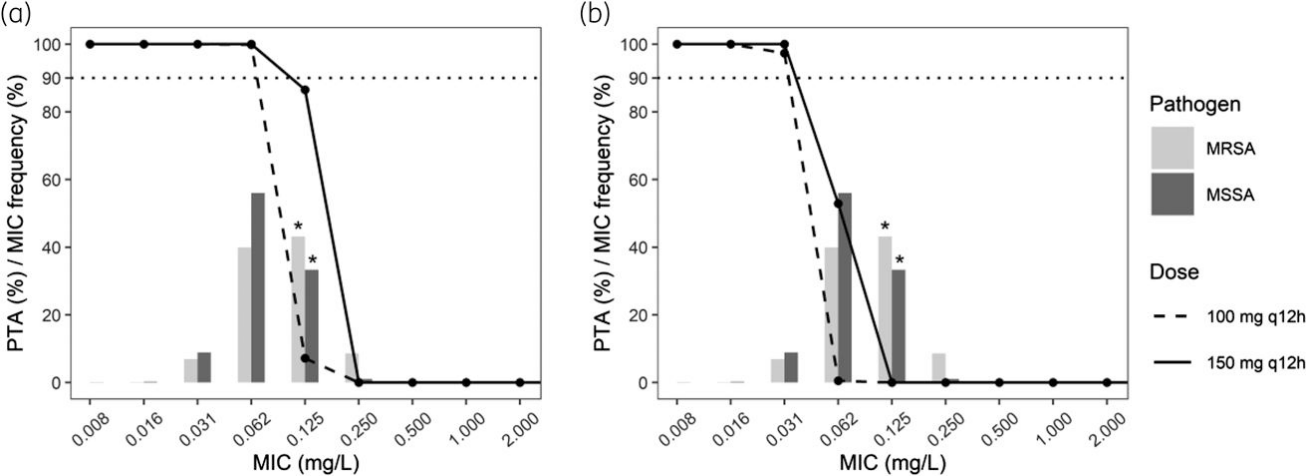
Probability of attaining the PK/PD targets associated with bacteriostasis (a) and a 1-log reduction in bacterial count (b) in a neutropenic murine thigh infection model for the 100 and 150 mg q12h regimens. The horizontal dotted lines indicate 90% PTA. The bars show the relative MIC distributions of MSSA and MRSA, and asterisks indicate the MIC_90_ values. Note: lefamulin MIC values >2 mg/L (*n* = 12 for MSSA, *n* = 21 for MRSA^9^) are not shown in this plot for the sake of legibility.

**Figure 2.**
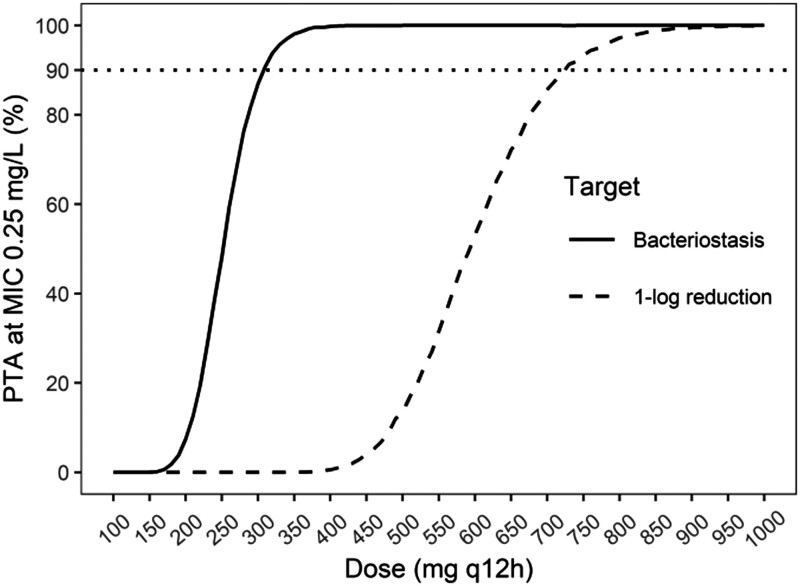
Probability of attaining the PK/PD targets associated with bacteriostasis and a 1-log reduction in bacterial count in a neutropenic murine thigh infection model at the ECOFF value of 0.25 mg/L for different lefamulin dosing regimens. The horizontal dotted line indicates 90% PTA. Note: doses >400 mg have not been administered in clinical studies of lefamulin.^10^

For the 150 mg q12h regimen, which is the licensed dose for CABP, the probability of attaining the bacteriostasis target was close to 90% at the *S. aureus* MIC_90_ of 0.125 mg/L (Figure 1). However, PTA was 0% at an MIC of 0.25 mg/L, the epidemiological cut-off (ECOFF) for *S. aureus*.^9^ In order to reach ≥90% PTA for the bacteriostasis target at the ECOFF, and thus cover the full WT distribution of *S. aureus*, the currently approved IV dose would have to be more than doubled (Figure 2), which is currently not sufficiently supported by safety data. In Phase 1 studies, single lefamulin doses ≥300 mg were associated with local intolerance.^10^ For the 1-log reduction target, PTA at the ECOFF was ≥90% only at doses untested in humans (Figure 2).^10^ Previous PTA analyses using plasma exposure targets derived from a neutropenic murine pneumonia infection model, used to support dose selection for the treatment of CABP, found that the 150 mg q12h IV regimen covers pathogens with MIC values up to 0.5 mg/L.^11^ The discrepancy with the results presented here can be explained by the lower exposure targets required for efficacy in the murine pneumonia model (a plasma *f*AUC_0–24h_/MIC of 2.13 to achieve a 1-log reduction in bacterial count for *S. aureus*), because lefamulin accumulates in epithelial lining fluid.^2,6^

Using the bacteriostasis target, the OTA against MSSA and MRSA was 68% and 50%, respectively, for the 100 mg q12h regimen, and 94% and 84%, respectively, for the 150 mg q12h regimen. When using the 1-log reduction target, the OTA against MSSA and MRSA dropped to 9% and 7% for the 100 mg q12h regimen, and 39% and 28% for the 150 mg q12h regimen. The PTA results thus correspond better with the positive results of a Phase 2 trial of lefamulin to treat ABSSSIs^3^ when using the bacteriostasis target than when using the 1-log reduction target. This potentially reflects that attaining PK/PD targets associated with bacteriostasis may be sufficient for treatment of ABSSSIs, as guidelines from the EMA also suggest.^12^ However, the low OTA for the 1-log reduction target suggests lefamulin may not be appropriate in situations where rapid bacterial killing is warranted, e.g. in severe ABSSSIs.

Our results indicate that the 150 mg q12h regimen offers substantially better OTA against *S. aureus* than the 100 mg q12h regimen. This is noteworthy given the absence of an apparent dose–response relationship in the aforementioned lefamulin trial.^3^ However, ABSSSI trials with limited numbers of patients may lack the sensitivity to reveal dose–response relationships, which could be due to several factors including a low number of treatment failures, spontaneous resolution of infections, subjective endpoint determination, and the impact of patient and pathogen factors on outcomes.^13,14^ Indeed, regulatory guidelines state that clinical dose-finding trials are not required for antibiotics if preclinical PK/PD analyses provide sufficient support for the dosing regimen selected for efficacy trials.^12^ The results presented here suggest that the 150 mg q12h regimen should be selected over the 100 mg q12h regimen in potential future trials evaluating lefamulin efficacy for the treatment of ABSSSIs, also considering the former was well tolerated in Phase 3 trials for the treatment of CABP.^15^

Several studies have compared antibiotics with respect to their OTA against *S. aureus*.^16–18^ The OTA is dependent on the underlying PK data, simulated populations and dosing regimens, PD endpoints and MIC distributions used in the analysis, which complicates comparisons between antibiotics and studies. Nonetheless, it is worth noting that the lefamulin OTA values found in this study were in some cases higher than the reported OTA for standard IV doses of other antibiotics with activity against MRSA used to treat ABSSSIs, such as vancomycin,^16–18^ linezolid^17,18^ and daptomycin.^18,19^ Only ceftaroline OTA values are markedly and consistently higher.^16,17^ Moreover, lefamulin resistance development and cross-resistance with other major antibiotic classes are infrequent,^1,19^ suggesting a potential role for lefamulin to treat infections that do not respond to first-line antimicrobial therapy.

In conclusion, this PTA analysis indicates that lefamulin at the IV dosage approved for CABP covers most *S. aureus* infections when targeting drug exposure associated with bacteriostasis, suggesting potential utility of lefamulin for the treatment of ABSSSIs, in particular in cases of resistance towards or contraindication against standard antimicrobial treatment options. Lefamulin may not be appropriate in ABSSSI when rapid bacterial killing is warranted.
